# Quantitative Seed Amplification Assay: A Proof-of-Principle Study

**DOI:** 10.1021/acs.jpcb.2c08326

**Published:** 2023-02-16

**Authors:** Jonathan Vaneyck, Therese A. Yousif, Ine Segers-Nolten, Christian Blum, Mireille M.A.E. Claessens

**Affiliations:** Nanobiophysics (NBP), Faculty of Science and Technology, MESA + Institute for Nanotechnology and Technical Medical Centre, University of Twente, PO Box 217, 7500 AE Enschede, Overijssel, The Netherlands

## Abstract

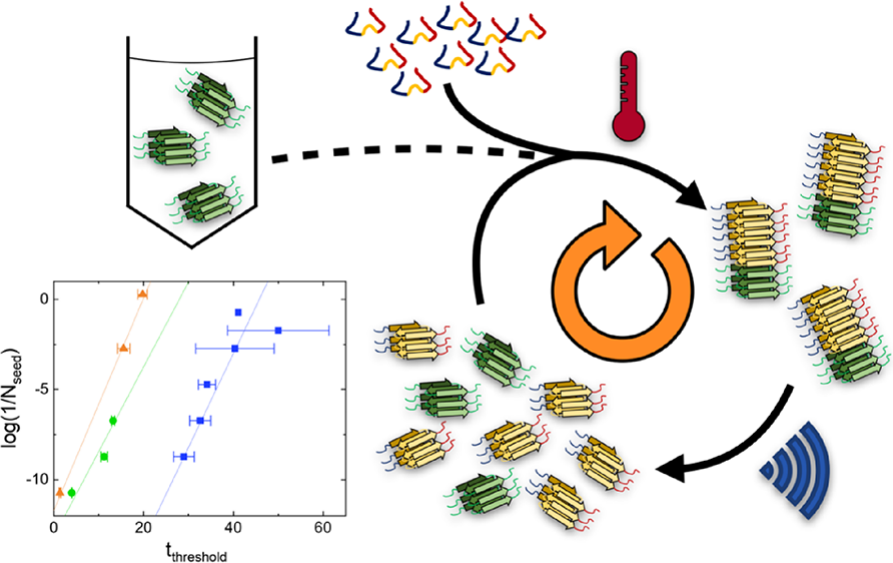

Amyloid fibrils of the protein α-synuclein (αS) have recently been identified as a biomarker for Parkinson’s disease (PD). To detect the presence of these amyloid fibrils, seed amplification assays (SAAs) have been developed. SAAs allow for the detection of αS amyloid fibrils in biomatrices such as cerebral spinal fluid and are promising for PD diagnosis by providing a dichotomous (yes/no) response. The additional quantification of the number of αS amyloid fibrils may enable clinicians to evaluate and follow the disease progression and severity. Developing quantitative SAAs has been shown to be challenging. Here, we report on a proof-of-principle study on the quantification of αS fibrils in fibril-spiked model solutions of increasing compositional complexity including blood serum. We show that parameters derived from standard SAAs can be used for fibril quantification in these solutions. However, interactions between the monomeric αS reactant that is used for amplification and biomatrix components such as human serum albumin have to be taken into account. We demonstrate that quantification of fibrils is possible even down to the single fibril level in a model sample consisting of fibril-spiked diluted blood serum.

## Introduction

Parkinson’s disease (PD) is the second most common and fastest-growing neurodegenerative disease.^1^ The clinical diagnosis of PD is based on symptoms, aided by imaging techniques like MRI and CT scans. PD diagnosis often remains ambiguous.^2,3^ A definitive diagnosis can only be achieved by a postmortem histological examination of brain tissue for the presence of Lewy bodies and Lewy neurites, which are deposits composed of amyloid fibrils of the protein α-synuclein (αS).

Under normal conditions, αS is abundantly present in the brain where it is thought to play a role in vesicle formation and trafficking.^4−9^ For reasons that are not well understood, αS self-aggregates in dopaminergic neurons of PD patients. This aggregation process is thought to result in cell death due to the formation of, e.g., toxic oligomers, membrane disruption by αS fibrils, or formation of intracellular amyloid aggregates.^10−14^

Recently, αS amyloid fibrils have been identified as a suitable biomarker for PD.^15^ αS amyloid fibrils have been found not only in brain tissue but also in other biomatrices like cerebral spinal fluid, skin, and blood to which they apparently leaked from the brain or from other tissues that share the αS pathology.^16^ The concentration of amyloid fibrils in these different biomatrices is however extremely low. To allow for the detection of such low concentrations of amyloid fibrils, seed amplification assays (SAAs) have been developed. Initially, the assays were developed for prion detection. Later, they proved to be of use for amyloids of other proteins found in other diseases.^17−19^

SAAs exhibit low experimental complexity and high specificity and sensitivity. In SAAs, the initial very low number of fibrils act as seeds for the amplification of the fibril mass to a detectable level. Amplification is achieved by adding non-aggregated monomeric αS as reactant to the sample of interest. The seeds in the sample will recruit monomeric αS reactant and elongate into longer fibrils. Shaking the sample induces fragmentation of the elongated fibrils, which increases the number of fibrils. These newly created fibrils again grow by recruiting monomeric reactant, and this cycle continues. This combination of events leads to an explosive increase in the number of fibrils and thus the fibril mass. In the SAA, the amplification is monitored using the fluorescent amyloid binding dye thioflavin T (ThT). Upon binding to the fibril, ThT becomes strongly fluorescent, which allows for the use of the total fluorescence intensity as a readout for the presence of fibrils once the seeds are sufficiently amplified. Although the SAA is in principle very sensitive to the presence of seeds, SAAs are only effective when de novo fibril formation is slow or inefficient compared to fibril amplification. De novo formation of fibrils can occur via several pathways. At sufficiently high αS reactant concentrations, nucleation can occur spontaneously. In addition, nucleation may also occur at interfaces depending on the experimental conditions.^20−22^ When these de novo processes are efficient, the assay is dominated by the amplification of de novo formed fibrils; the presence of seeds only plays a minor role. The sensitivity of the SAA is hence limited by de novo fibril formation. The presence of seeds in a sample is visible as a shortening of the time required to reach a threshold ThT intensity compared to a control sample obtained under identical conditions in which seeds are absent.

SAAs have been used to detect the presence of αS seeds in different biomatrices including cerebrospinal fluid and skin obtained from PD patients, and their applicability to other biomatrices is being explored in several laboratories.^19,23−30^ Currently, the outcome of SAAs is used to determine whether seeds are present or not, providing a dichotomous response. Quantitative SAAs, in which the number of seeds in a sample is determined, would be a step toward enabling clinicians to monitor the effect of treatments, compare patients, and make predictions on disease progression. However, for a quantitative assay, it would be necessary to calculate back the initial seed concentration.

First steps toward developing quantitative SAAs have been taken by using end-point dilution assays in the plate reader or by using digital microfluidics.^31^ Additionally, the relation between seed dilution and the timepoint at which half of the maximum ThT fluorescence is reached was explored. Especially in compositionally more complex biomatrices, quantification has proven challenging.^29,32^ One of the complicating factors in these complex biomatrices is presumed to be the interaction of reactant αS with matrix components.^33^

For αS, we have shown earlier that, at neutral pH and under agitation, fibril growth can be described taking into account only fibril breaking and monomer addition.^34^ This enables quantification as the combined effect of both contributions to the amplification can be modeled. Here, we show in a proof-of-principle study under controlled conditions that it is possible to use the time to reach a threshold ThT intensity (*t*_threshold_) to make αS SAAs quantitative. We performed experiments in model solutions of increasing compositional complexity, including blood serum, spiked with known amounts of αS seeds (*N*_seed_). We measured the seed length distribution to determine the *N*_seed_ used for spiking. As for all conditions tested, the measured *t*_threshold_ scales with *N*_seed_; this relation allows for quantification via a reference curve. We show that it is possible to approach the single seed level in quantification. We identified human serum albumin (HSA) as the main interaction partner of reactant αS and hence the major complicating factor in quantification of SAAs in blood serum. Determining the αS–HSA interaction strength allowed us to shift the equilibrium toward free reactant αS by diluting the serum, which restored the seed amplification.

## Materials and Methods

### Expression and Purification of αS

αS was produced recombinantly, as described by Sidhu et al.^48^ Briefly, αS was produced in *Escherichia coli* (*E. coli*) cells transformed with the pT7–7 plasmid carrying the αS gene. αS production was induced using IPTG. Finally, *E. coli* cells containing the protein were lysed and αS was purified by standard methods. Aliquots of αS were stored at -80 °C.

### Sampling of Human Serum

SiO_2_-coated 9 mL tubes (Vacuette, Prod. No. 455092) with each 9 mL of whole blood were requested and obtained from the TechMed Centre donor service (University of Twente, Enschede, the Netherlands). Informed consent was obtained from the donors, and the study was approved by the TechMed Centre donor service. The tubes containing blood were left for 24 h in the fridge at 4 °C before they were centrifuged for 10 min at 1000 × *g* at 4 °C. The supernatants (serum) were pooled. 1 mL aliquots of the serum were stored in 1.5 mL tubes at −20 °C until needed for further use.

### Production of αS Amyloid Fibril Seeds

Recombinantly produced αS monomers were thawed and filtered through an Anotop 10 mm Whatman 0.02 μm filter. The protein concentration was determined by measuring the absorbance at 280 nm and using a molar extinction coefficient of 5600 M^–1^ cm^–1^. This solution of αS was diluted to 100 μM in 10 mM Tris, 10 mM NaCl, and 0.1 mM EDTA at pH 7.4. The αS solution was incubated in low-bind round-bottom Eppendorf tubes at 37 °C in an Eppendorf Thermomixer Comfort at an orbital shaking speed of 750 rpm for 1 week. After this incubation time, αS had aggregated into amyloid fibrils. For further use, the tubes were pooled and centrifuged at 21,000 × *g* for 1 h at room temperature. The supernatant was used to determine the residual monomer concentration by measuring the absorbance at 280 nm. The pellet was resuspended in a fixed volume, and the fibril concentration (monomer equivalent) was determined considering the residual monomer concentration. The fibril solution was stored at room temperature. To induce fragmentation and produce seeds, the fibrils were sonicated in a Branson 2510 Ultrasonic Cleaner bath sonicator (Branson Ultrasonics Corp., Brookfield, Connecticut, USA).

### Determination of the Length of the αS Seeds by Atomic Force Microscopy (AFM)

20 μL of the seed solution was placed on top of a freshly cleaved mica disk (muscovite mica, V-1 quality EMS) and incubated for 4 min. The mica was rinsed three times with demineralized H_2_O to remove unbound seeds before the sample was dried with a soft flow of nitrogen (N_2_) and stored overnight at room temperature. AFM images were acquired using a bioscope Catalyst (Bruker, Santa Barbara, California, USA) in soft tapping mode in air using the NSC36 tip B probe (MikroMasch, Tallin, Estonia) with a force constant of 1.75 N/m. Images were acquired with a maximum scan size of 10 μm × 10 μm, a scan rate of 1.0 Hz, and a resolution of 512 pixels/line. Images were post-processed using the Gwyddion software package (2018, version 2.55). The seed length distribution was determined by measuring the length of the individual fibrils using Scanning Probe Image Processor-6.0.13 (SPIP; Image Metrology) software.

### SAA

SAAs were performed in two different buffer conditions, one of low ionic strength (10 mM Tris, 10 mM NaCl, 0.1 mM EDTA, pH 7.4) and another of high ionic strength (10 mM Na_2_HPO_4_, 1.8 mM KH_2_PO_4_, 137 mM NaCl, 2.7 mM KCl, pH 7.4). The αS reactant was filtered through an Anotop 10 mm Whatman 0.02 μm filter, and the protein concentration in the SAA was set to 22 μM. The samples were spiked with preformed seeds at the equivalent monomer concentrations given in the main text. To monitor seed amplification, a total of 22 μM of ThT was added. The experiments were performed in 96-well polystyrene microplates (Nunc, Prod. No. 655096) and covered with a seal (Nunc, Prod. No. 676070). The sample volume was 200 μL, and the experiments were performed in quadruplet. Seed amplification was monitored for 1000 cycles at 37 °C with each cycle consisting of five repetitions of 1 min of orbital shaking at 355 rpm and 1 min of no shaking in a plate reader (Infinite M200 PRO fluorescence plate reader, Tecan). The fluorescence intensity (excitation and emission wavelength of 458 and 485 nm, respectively) was measured from the bottom of the plate at the beginning of each new cycle with a gain setting of 100 to obtain αS aggregation curves for each individual well.

To quantify seed amplification, we calculated the time-averaged aggregation curves for each seed concentration and experimental condition. The individual aggregation curves were normalized to the plateau ThT intensity values. For every 1% intensity increase, we determined the nearest timepoint in each individual aggregation curve. We use this new data set to determine the mean time and the standard deviation for each intensity value.

The SAAs were also performed in human serum and diluted human serum following the same protocol. By adding αS reactant and seeds, the serum was diluted by 10%. For the experiments in diluted serum, serum was diluted 70 times in the high ionic strength buffer (10 mM Na_2_HPO_4_, 1.8 mM KH2PO4, 137 mM NaCl, 2.7 mM KCl, pH 7.4) before adding αS reactant and seeds.

### Microscale Thermophoresis (MST)

The affinity between HSA and Alexa Fluor 488 labeled αS (αS-AF488) was determined using a Monolith NT.115 (NanoTemper Technologies GmbH, Munich, Germany) MST system. The thermophoretic movement was monitored using a constant concentration of αS-AF488 (50 nM) with a dilution series of 16 different concentrations of HSA (from 50 μM to 6.25 nM) prepared in high salt buffer (10 mM Na_2_HPO_4_, 1.8 mM KH_2_PO_4_, 137 mM NaCl, 2.7 mM KCl, pH 7.4). Capillaries (MO-K002, standard treated, NanoTemper Technologies GmbH, Munich, Germany) were filled with the different samples. A capillary scan was made to ensure that the initial fluorescence intensity was the same in each of the capillaries. The MST was measured using a LED power of 20% and a MST power (IR laser) of 20, 40, and 80%. The plateaus in the binding curves obtained at the different MST powers were used to normalize the data. For each concentration, the data was averaged.

## Results

**Figure 1:**
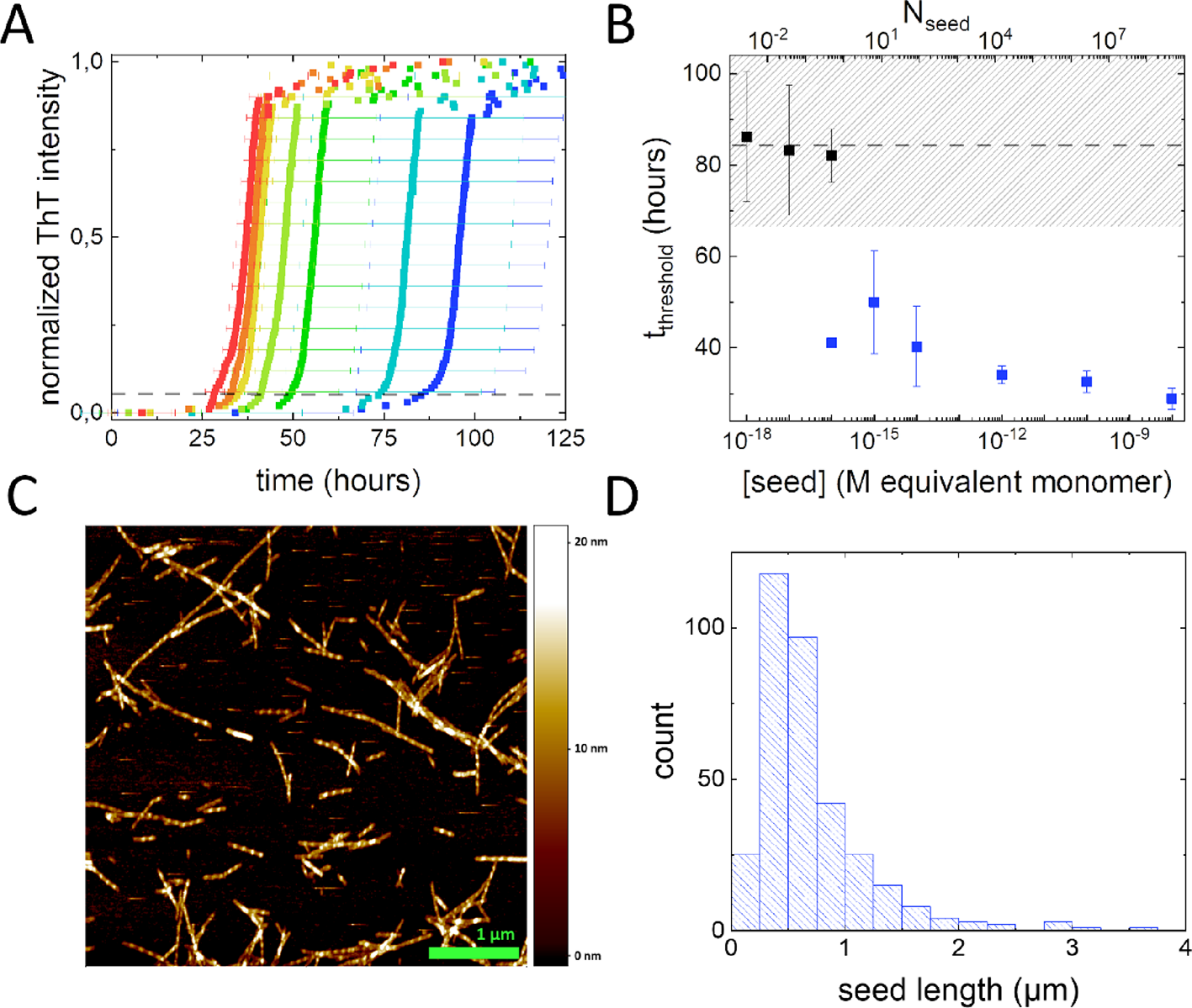
SAA performed in a low salt buffer solution and characterization of seeds. (A) Selection of SAAs at increasing seed concentration monitored using ThT fluorescence in a simple low salt buffer (10 mM Tris, 10 mM NaCl, 0.1 mM EDTA, pH 7.4), and 22 μM reactant αS. A rainbow color coding is used to depict different seed concentrations with red representing a high seed concentration and blue a low seed concentration (10^–8^, 10^–10^, 10^–12^, 10^–14^, 10^–15^, 10^–16^, and 10^–17^ M equivalent monomer seed concentrations). The data represents normalized time averaged quadruplicates. The error bars represent the standard deviation on the data. (B) The evolution of the threshold time with increasing seed concentration. The threshold time, *t*_threshold_, was determined at 5% of the plateau ThT intensity from individual curves. The data is shown for both the monomer equivalent seed concentration (bottom *x*-axis), and the number of seeds present in the 200 μL sample volume (top *x*-axis). The dashed black line and shaded area indicate the *t*_threshold_ and standard deviation observed for the control sample containing no seeds. For *t*_threshold_ comparable to the control without seeds the data points are shown in black. For *t*_threshold_ indicating the presence of seeds data is shown in blue. (C) Typical atomic force microscopy (AFM) image of the αS fibrils used as seeds in the SAAs. Sample height is color coded as indicated by the vertical bar. (D) Length distribution of the seeds obtained from the AFM images. The median seed length obtained from this length distribution was used to calculate *N*_seed_ from the monomer equivalent seed concentrations.

In a first step, we investigated how the αS seed concentration affects the αS SAA in a simple, low salt buffer. For the SAAs, a solution of monomeric reactant αS was spiked with αS seeds at eight different concentrations, ranging from 10^–8^ to 10^–18^ M equivalent αS monomer concentrations. Additionally, we followed the spontaneous aggregation of αS in a control sample containing no seeds. We used ThT fluorescence intensity as a readout for the increase in αS fibril mass in the sample. For all the samples, the ThT intensity increased over time. A selection of the typical normalized time-averaged αS aggregation curves is plotted in Figure 1A. For samples in which seeds are absent, αS aggregation is detected at timescales >70 h for all replicates. With increasing seed concentration, the ThT curves shift to shorter timescales and the spread in time within the quadruplicates decreases. At the highest seed concentration used, an increase in ThT fluorescence is detected at around 30 h and the data from the quadruplicate agrees well.

To quantify the seed concentration dependence of the SAA, we determined a threshold time from the aggregation curves. This threshold time is defined as the time required to reach a defined fraction of the final, plateau ThT intensity detected in the well. The threshold times are selected to be in a regime where the fibril mass increases exponentially with time. In this series of experiments, we set the threshold to 5% of the plateau ThT intensity. For control samples containing no seeds, we find an average time to the threshold (*t*_threshold_) of around 84 ± 20 h. This implies that threshold times in this range are the result of de novo fibril formation and not of seed amplification. In analyzing the dataset, we therefore differentiate between *t*_threshold_ < 64 h and *t*_threshold_ > 64 h, representing the regimes in which the presence of seeds can and cannot be detected. In Figure 1B, the average *t*_threshold_ is plotted as a function of the equivalent monomer concentration; the regime in which seeds cannot be detected is shaded. For equivalent monomer concentrations >10^–15^ M, we consistently find a *t*_threshold_ < 64 h, evidencing the presence of seeds. For equivalent monomer concentrations <10^–17^ M, we consistently find a *t*_threshold_ > 64 h, indicating that the assay is dominated by de novo fibril formation at these equivalent monomer concentrations. At an equivalent monomer concentration of 10^–16^ M, three of the four replicates have a *t*_threshold_ > 64 h and one has a *t*_threshold_ < 64 h. The average of *t*_threshold_ > 64 h and the single data point for *t*_threshold_ < 64 h are shown separately in Figure 1B. With increasing seed concentrations, both *t*_threshold_ and the relative spread on the data decrease. The observations qualitatively match expectations. At very low equivalent monomer seed concentrations, the reaction volume contains only a low number of seeds. At these low numbers, the amplification of de novo formed fibrils may dominate over the seed amplification. In this case, *t*_threshold_ will remain constant and comparable to *t*_threshold_ in the control samples. The spread of the data at very low seed concentrations represents the stochasticity of the de novo fibril formation. With increasing seed concentration, the contribution of de novo formed fibrils to the amplification decreases, resulting in a decrease in *t*_threshold_ since fewer rounds of amplification are needed to obtain the fibril mass required to reach the threshold ThT fluorescence.

To go beyond qualitative agreement and to quantify *N*_seed_, it is necessary to translate the equivalent monomer concentrations to the concentration of seeds. When fibril breaking and monomer addition are the mechanisms responsible for seed amplification, the amplification does not scale with the total seed mass initially present in the sample but with the number of seed ends. In the SAA, the fibril mass increases due to extension of the seeds at their ends followed by fragmentation of the grown fibrils to create more fibril ends. This process of fibril elongation and fragmentation will lead to an exponential increase in fibril mass that can be observed as an increase in ThT signal. To determine the number of seeds and hence the number of seed ends that was initially present in the sample, we determined the length distribution of the seeds used. To do so, we deposited samples of seeds on freshly cleaved mica and recorded AFM images. In the images (Figure 1C), seeds of different length are clearly visible. From the images, we determine the length distribution of the seeds (Figure 1D). We observe a wide distribution of seed lengths, which peaks around 0.4 μm and extends up to 3.8 μm. The median length of the αS seeds is 0.57 μm. We subsequently used this median length to convert the equivalent monomer seed concentration to the number of seeds in the sample volume. In this conversion, we assumed that the seeds are double-stranded fibrils with a β-strand distance of 0.5 nm.^35^ Based on these assumptions, we estimated that the median seed contains approximately 2300 αS monomers. We converted the equivalent monomer seed concentration to the number of seeds in the sample and added *N*_seed_ as an additional axis to Figure 1B. From Figure 1B, it becomes clear that the 200 μL samples need to contain ≥5 seeds to distinguish samples that contain seeds from those that do not. In samples containing effectively no seeds, the amplification of de novo formed fibrils dominates. With an increasing *N*_seed_ to values above approximately 5, *t*_threshold_ decreases. At low *N*_seed_, the standard deviation of *t*_threshold_ is still large due to the competition between fibril growth from seeds and fibril growth from de novo formed fibrils. Note that in our data we see the stochastic occurrence of seeds for *N*_seed_ ≈ 0.5 where only one out of four replicates resulted in seed amplification. Our data confirms that the SAA is very sensitive; in a simple low salt buffer, detection is possible approaching the single seed level.

**Figure 2:**
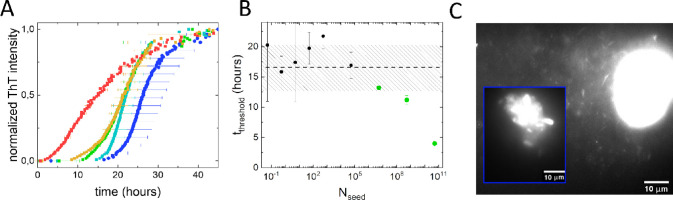
SAA performed at physiological salt conditions. (A) Selection of SAAs performed in PBS (10 mM Na_2_HPO_4_, 1.8 mM KH_2_PO_4_, 137 mM NaCl, 2.7 mM KCl, pH 7.4) at increasing seed concentrations monitored using ThT fluorescence at a reactant αS concentration of 22 μM. A rainbow color coding is used to depict different seed concentrations with red representing a high seed concentration and blue a low seed concentration (10^–6^, 10^–8^, 10^–10^, 10^–12^, and 10^–15^ M equivalent monomer seed concentrations). The error bars represent the standard deviation on the time-averaged data. (B) *t*_threshold_ decreases with increasing *N*_seed_. The dashed black line and shaded area indicate the *t*_threshold_ and standard deviation observed for the control sample that did not contain any seeds. For *t*_threshold_ comparable to the control that did not contain seeds, the data points are shown in black. Data points that indicate the presence of seeds are shown in green. (C) Fluorescence microscopy image of fluorescently labeled αS seeds showing seed clumping at high salt concentrations. This clumping is responsible for the low sensitivity of the SAA at these buffer conditions (B). The inset shows the large aggregate on the right with at a different intensity scale.

In a next step, we performed SAAs at higher ionic strengths representing the physiological salt concentrations found in patient samples. As above, samples containing a range of seed concentrations were made and the seed amplification was followed in a ThT αS aggregation assay. In Figure 2A, we show a selection of the averaged ThT curves obtained. Compared to the low ionic strength conditions, *t*_threshold_ is reached considerably faster at higher ionic strength. This is in agreement with literature on the ionic strength dependence of αS aggregation.^36^ From the aggregation curves, we again determine *t*_threshold_ (5% of plateau ThT intensity) and plot this as a function of *N*_seed_ present in the sample (Figure 2B). To calculate *N*_seed_, we again used the median seed length determined from Figure 1D. Once more, we observe that at low *N*_seed_, *t*_threshold_ is comparable to *t*_threshold_ of the control, while at higher *N*_seed_, *t*_threshold_ decreases. Again, there is a clear concentration dependence of *t*_threshold_ on *N*_seed_. However, compared to low ionic strength conditions, the SAA in physiological salt conditions is much less sensitive. The sample has to contain a very large number of αS seeds before amplification of the initially added seeds becomes dominant. The drop in sensitivity may be assigned to the more efficient de novo fibril formation at higher ionic strength that masks seed amplification.^36^ In addition, fluorescence microscopy images of fluorescently stained seeds at high ionic strength show clear signs of seeds clumping together (Figure 2C). This clumping is in agreement with previous observations^37^ and will also affect the SAA, as, in the clumps of seeds, many fibril ends are buried and hence non-accessible for elongation. Additionally, the interactions between fibrils in the clump stabilize the fibrils against fragmentation and this hampers the amplification of the number of fibril ends. The presence of seeds is evidenced not only by the drop in *t*_threshold_ with *N*_seed_ but also by the strong decrease in the standard deviation of *t*_threshold_.

**Figure 3:**
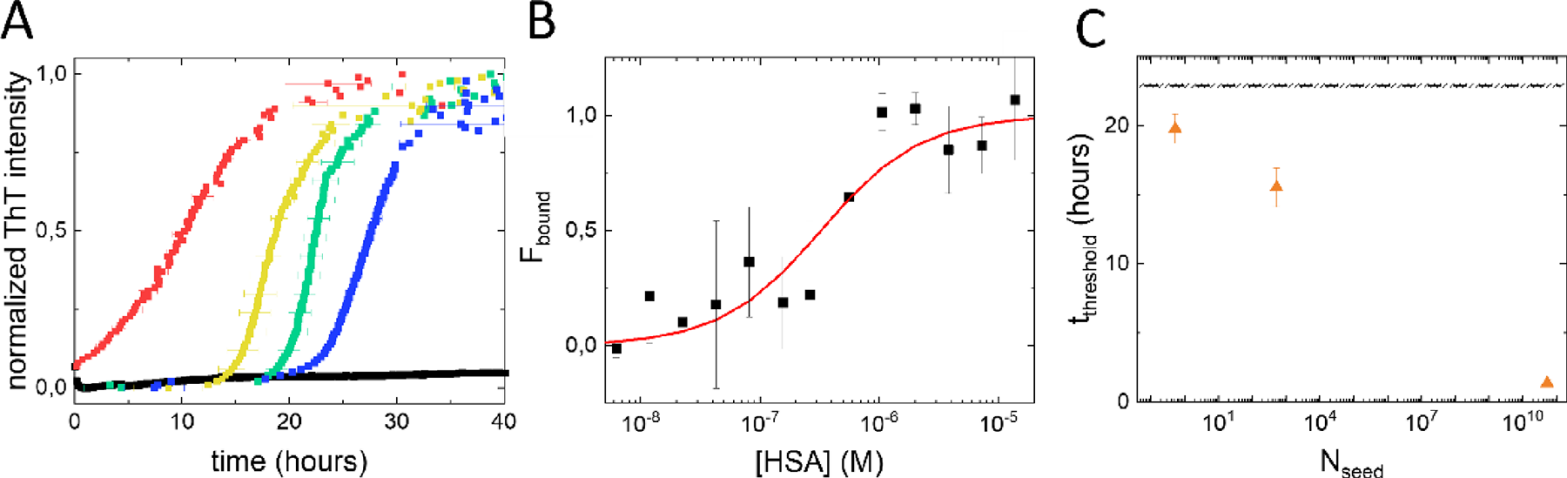
SAAs in blood serum and interaction between αS and HSA. (A) We do not observe any seed amplification in undiluted blood serum (black, 10^–6^ M equivalent monomer seed concentration). Diluting the serum 70 times restores the seed amplification, rainbow color-coded data with blue representing the absence of seeds (control sample) and red a high seed concentration (0, 10^–17^, 10^–14^, and 10^–6^ M equivalent monomer seed concentration, respectively). (B) Interaction between αS and HSA monitored using microscale thermophoresis depicted as the fraction of αS bound (*F*_bound_). In the experiment, the concentration of fluorescently labeled αS was kept constant while the HSA concentration was increased. The red line represents simple equilibrium binding between αS and HSA with an approximate *K*_D_ of 300 nM. (C) The *t*_threshold_ decreases with increasing *N*_seed_. The dashed black line and shaded area indicate *t*_threshold_ and standard deviation observed for the control sample containing no seeds.

Since there have been reports that αS aggregates in blood serum can potentially serve as a biomarker for Parkinson’s disease, we tested the SAA in this easily accessible biomatrix.^38^ However, when we performed the SAA in spiked full blood serum, using the same concentration of reactant αS, we did not observe any αS aggregation on the time scale of the experiment (∼190 h, not fully shown, black line in Figure 3A). Since αS aggregates faster at high ionic strength, the high ionic strength in serum cannot be the cause of the observed effect. Interactions between the reactant αS and blood components potentially interfere with the SAA. HSA is the most abundant protein in serum. In literature, the presence of serum albumin has been reported to impede αS aggregation.^39^ With HSA concentrations in serum ranging 35–50 g/L or 0.5–0.75 mM, HSA may (aspecifically) interact with αS. We used an MST assay to investigate the interaction between HSA and αS and to quantify the binding affinity. In Figure 3B, we show that the two proteins interact with an apparent equilibrium dissociation constant of *K*_D_ ≈ 300 nM. With this affinity and a reactant αS concentration of 22 μM, approximately 99.9% of the αS is bound to HSA and not readily available for seed elongation. One way to address this problem is by diluting the serum while keeping the αS reactant concentration constant. To have at least half of the reactant αS directly available for seed amplification, we diluted the samples 70 times. With a *K*_D_ ≈ 300 nM and a reactant αS concentration of 22 μM, diluting the serum 70 times results in a decrease of the fraction of reactant αS bound to HSA to approximately 40%. In serum that was 70 times diluted with PBS, αS aggregation restores as evidenced by the increase in ThT signal with time (Figure 3A). Once more, we see a clear seed concentration dependence of the SAA. With increasing αS seed concentration, *t*_threshold_ decreases. The αS aggregation is fast and the spread of the αS aggregation curves is low. De novo αS fibril formation appears to be very reproducible under these conditions. Plotting *t*_threshold_ as a function of *N*_seed_ (Figure 3C), determined as described above, shows that the assay is very sensitive; a very low number of seeds can be detected in this SAA.

**Figure 4:**
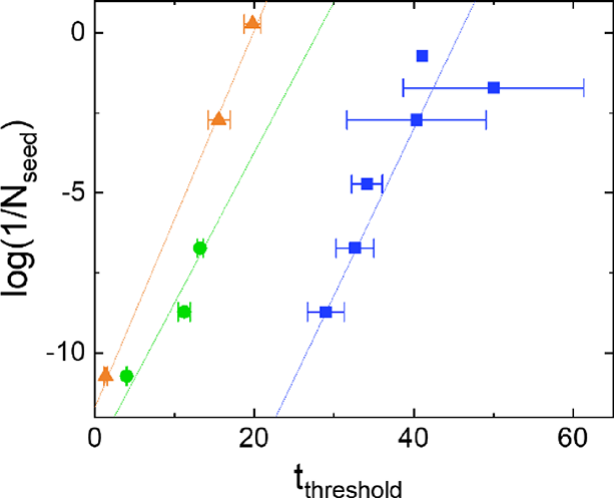
Relation between l and *t*_threshold_. Plotting log(1/*N*_seed_) as a function of *t*_threshold_ for the experiments under different conditions shows a linear relation between the two parameters evidencing a constant doubling time *t*_D_. In this graph, only seed concentrations for which seed amplification was observed are included. The lines serve as guide to the eye to visualize the linear relation. Data for the SAA in low salt buffer is shown in blue, in PBS in green, and in diluted blood serum in orange.

The αS SAAs rely on fibril elongation and fragmentation. In the ideal case, de novo fibril formation is negligible and the fragmentation and elongation rates are constant. In this case, the fibril mass doubling time is constant. This can be expressed as , where *N*(*t*) is the number of fibrils at the timepoint *t* and *t*_D_ is the doubling time. At the time *t*_threshold_, a fixed (but unknown) number of seeds, *N*_threshold_, will be present in the sample. The expression can therefore be rewritten as . The expression shows that the relation between the added *N*_seed_ and the observed *t*_threshold_ scales as  and that the relation between  and *t*_threshold_ is linear for constant *t*_D_. In Figure 4, we plot  as a function of *t*_threshold_ for all three conditions tested. We include *N*_seeds_ for which we observe amplification as evidenced by a shortened lag time. Seed amplifications in low (blue) and high salt buffer (green), as well as in diluted serum (orange), show the expected linear relation. For all tested model solutions, *t*_D_ is constant and hence independent of *N*_seed_. The observed linear relation evidences that the contribution from de novo fibril formation to the amplification can be neglected for the range of seed concentrations shown and the experimental conditions used.

## Discussion and Conclusions

Our proof-of-principle study shows that SAAs performed in standard fluorescence plate reader experiments can provide quantitative results down to the single fibril level for the well-defined conditions tested here. In the experiments, we observed a linear relation between *t*_threshold_ and log(1/*N*_seed_) in the regime where seed amplification dominated over the amplification of de novo formed fibrils. With this linear relation, quantification of *N*_seed_ in samples of interest is possible. However, the single seed level was not reached for all the conditions tested, e.g., due to efficient de novo fibril formation or inter-fibril interactions. An additional complication that needed to be considered is the interaction of reactant αS with biomatrix components, such as HSA in blood serum, which reduces the amount of reactant αS available for seed amplification. We show that simple dilution of blood serum restored seed amplification by shifting the binding equilibrium between αS and the αS–HSA complex to free reactant αS. An alternative approach to ensure that there is sufficient αS reactant available might be increasing the αS reactant concentration. This approach would however consume large amounts of αS and make the SAA more costly. A possible advantage of increasing the reactant αS concentration over sample dilution is that it likely does not suffer from the decrease in sensitivity that comes with dilution of the sample. However, higher αS reactant concentrations facilitate de novo fibril formation, which negatively affects the sensitivity of the SAA.

Although the assay can be extremely sensitive, the question remains if this sensitivity can match the seed concentrations that may be present in patient material. For the experiments in diluted blood serum, our data indicates that in quantification the single fibril level can be approached. For a sample of 200 μL to contain five fibrils with a median length of 0.57 μm (Figure 1C), approximately only one neuron (volume ≈ 12,260 μm^3^, [αS] ≈ 20–40 μM) has to leak its full αS content in the form of seeds into the blood stream. Performing an SAA on diluted samples effectively shifts the sensitivity of the SAA to higher initial seed numbers, in our case 70 times. The number of αS seeds in the bloodstream is however most likely higher than the five fibrils per 200 μL mentioned above. Aggregation competent αS species are continuously released from cells, and seeds probably not only originate from the brain but also from other tissues that are autonomously innervated and share the αS pathology.^16,40^

To establish the sensitivity of the SAA and to convert the measured *t*_threshold_ to *N*_seed_ in a patient sample, a reference curve is required. The data shown in Figure 4 can serve as reference curves for the three different conditions tested. For all the conditions tested, there was a linear relation between *t*_threshold_ and log(1/*N*_seed_), but the offset between the conditions differs. The nature of the differences in offset is currently unknown. The presence of the offset highlights the need for reference curves obtained under the same experimental conditions instead of relying solely on measured doubling times, *t*_D_. Due to compositional differences between different biomatrices, it will be necessary to optimize and obtain reference curves for each biomatrix. We do however not expect that for a given biomatrix, patient-to-patient differences in composition will play a major role. Small patient-to-patient differences in matrix composition are expected but are unlikely to play an important role in the SAA by, e.g., changing the available reactant αS concentration. It is worth noting that there are compounds that can induce aggregation of reactant αS and the presence of these compounds in patient samples would interfere with the SAA.^41−43^

In blood serum, the presence of αS from red blood cells is a concern. However, αS concentrations in blood plasma have been reported to be in the order of 25 ng/mL or equivalently 2 nM.^44^ In red blood cells, the αS concentration is of the order of 26,200 ng/mL, which is approximately 2 μM.^22,23,44,45^ Even if red blood cells leak αS into serum, the αS concentration will be low compared to the added reactant αS. We therefore expect that red blood cell αS does not affect the assay in (diluted) blood serum.

Although the presented quantification of αS seed numbers is promising, the SAA can be further improved. Quantification in the presence of de novo fibril formation remains a challenge especially for low seed numbers. In our experiments, de novo fibril formation is most likely mediated by surfaces; we expect that it can be further suppressed by using low bind microplates. Alternatively, de novo fibril formation can be slowed down or completely suppressed by using the αS K23Q mutant as the αS reactant.^26^ A well-defined aggregation lag time in absence of seeds, as we observe for experiments in diluted serum, is beneficial for the quantification of low seed numbers. Why de novo fibril formation is so well defined in diluted serum is unknown. Potentially interactions with serum components limit the configurational freedom of αS, leading to a better defined nucleation of new fibrils. Additives such as low concentrations of SDS may increase the accuracy of the SAA by this mechanism.^46,47^

In summary, our proof-of-principle study shows the potential of SAAs for the quantification of seed concentrations, even in compositionally complex samples. The sensitivity of the SAA is limited by the de novo formation of fibrils. However, for a broad range of *N*_seed_, the amplification is not hampered by de novo formation of fibrils; for these *N*_seed_, we find a constant doubling time for all solution conditions tested. We show that under controlled conditions, seed quantification is possible down to the single seed level via a reference curve in a standard fluorescence plate reader assay. In compositionally more complex biomatrices, interactions with reactant αS interfere with the assay but relatively simple measures, like a dilution step, can restore αS seed amplification. Our study demonstrates the potential of SAAs for fibril seed quantification, but at the same time, it indicates that inhomogeneity of the seed population, compositional differences of the biomatrices, and interactions between biomatrix components and reactant αS remain a challenge.
